# Supplementary material for: Analysis on urban scaling characteristics of China’s relatively developed cities
Source: PLoS One. 2020 Jul 29;15(7):e0236593. doi: 10.1371/journal.pone.0236593 (PMC7390399; doi:10.1371/journal.pone.0236593)
Supplement: S1 Table — (DOC) [file pone.0236593.s001.doc]

**S1 Table. Detailed information of various indicators.**

| **Abbreviation** | **Variable** | **Unit** | **Category** | **Source** |
| --- | --- | --- | --- | --- |
| POP | Population | Ten thousand people | Base | CCSY |
| GRP | Gross regional product | Ten thousand yuan | Social innovation wealth/Base | CCSY |
| TGS | Total gas supply | Ten thousand cubic meters | Individual demands | CCSY |
| TWS | Total water supply | Ten thousand tons | Individual demands | CCSY |
| WSEC | Whole society electricity consumption | Ten thousand kW-h | Individual demands | CCSY |
| FAI | Fixed asset investment | Ten thousand yuan | Individual demands | CCSY |
| CLA | Construction land area | Square kilometers | Individual demands | CCSY |
| DPL | Drainage pipe length | Kilometers | Individual demands | CUCSY |
| RA | Road area | Ten thousand square meters | Urban infrastructure | CUCSY |
| PA | Park area | Square meter | Urban infrastructure | CUCSY |
| GCA | Green coverage area | Square meter | Urban infrastructure | CUCSY |
| SLN | Street lamp number | Number | Urban infrastructure | CUCSY |
| TNGS | Total natural gas supply | Ten thousand cubic meters | Individual demands | CCSY/CUCSY |
| TGS-LPG | Liquefied petroleum gas | Ton | Individual demands | CCSY/CUCSY |
| EU | Electricity use | Ten thousand kW-h | Individual demands | CCSY |

**Note:**

**CCSY:** China City Statistical Yearbook;

**CUCSY:** China Urban Construction Statistical Yearbook;
