# Supplementary material for: Analysis on urban scaling characteristics of China’s relatively developed cities
Source: PLoS One. 2020 Jul 29;15(7):e0236593. doi: 10.1371/journal.pone.0236593 (PMC7390399; doi:10.1371/journal.pone.0236593)
Supplement: Attachment — Submitted filename: Response to Reviewers.doc [file pone.0236593.s002.doc]

Dear Editors and Reviewers:

Thank you for your letter and for the reviewers’ comments concerning our manuscript entitled “Analysis on urban scaling characteristics of China’s relatively developed cities” (ID: PONE-D-20-08745). Those comments are all valuable and very helpful for revising and improving our paper, as well as the important guiding significance to our researches. We have studied comments carefully and have made correction which we hope meet with approval. Revised portions are marked in the paper.

The main corrections in the paper and the responds to the reviewer’s comments are as following.

**Responds to the reviewer’s comments:**

**Reviewer #1:**

**General comments:
1. 114 cities are not a decent sample size, especially when the sample is further classified into three groups. At such small sample size, especially the 14 industrial cities, how do you make sure the regression results are robust?**

**Response:**

The number of cities is not an important factor affecting the regression results. It can be found from Table 2 that the number of commercial cities is only 27, and the number of mixed-economy cities is 73. However, the regression effect of commercial cities is significantly better than that of mixed economy cities, which to some extent indicates that the number of different types of cities will not affect the reliability of the results. In fact, we have also calculated the scaling index of different urban development indicators with urban population and urban GRP under more industrial cities (including the fourth-tier and below industrial cities, a total of 42), and the results are generally consistent with the results of this study, so I think that even if the number of industrial cities is relatively small, it will not have a big impact on the reliability of the regression results. It is the great difference between different industrial cities that leads to the less obvious scaling characteristics of industrial cities.

**2. Why use supply instead of gas and water consumption data while the electricity consumption data is used? Are there factors such as import of gas, water resource allocation would affect supply and consumption?**

**Response:**

The total amount of urban gas supply refers to the total amount of gas supplied by gas enterprises to users during the reporting period, including the amount of sales and losses. Total urban water supply refers to the total amount of water supplied by water supply units during the reporting period, including effective water supply and leakage water. Leakage of gas and water mainly refers to the leakage caused by damage to pipelines and ancillary facilities, theft or failure of counting tables in the process of water supply. Leakage can help us judge whether the construction of urban infrastructure has been improved to some extent with the expansion of city scale. Therefore, the total water supply and gas supply were used in our study, rather than the consumption index after the leakage was removed. Urban power loss is usually caused by electricity transportation, which is an inevitable quantitative technical loss. Therefore, the city statistical yearbook usually only collects electricity consumption data and does not include electricity loss data. Therefore, we also use electricity consumption data instead of electricity supply data in our research.

**3. A table showing more details of the data used in the study would help the readers to know better about your research.**

**Response:**

According to your suggestion, we have made a table to help readers understand the specific information of the research indicators, including the indicator name, unit, specific classification and data source. This table has been included in the supporting information.

**4. I don’t think park area is a good example for urban infrastructure, especially when explaining its sub-linear relationship with population or GRP.**

**Response:**

Urban park is an important part of China's urban construction planning, and the park area has always been an important indicator to measure the level of China's urban modernization. The park plays an important role in helping to improve the city's ecological environment and residents' living environment. Therefore, we believe that park area should be an important part of urban infrastructure.

Of course, we carefully considered your opinion and decided to increase the "green area coverage" as one of the urban infrastructure indicators. The urban green space coverage area covers a wider range. In addition to the park green area, it also includes the city's residential green space and traffic green space, so it may be a better indicator of urban infrastructure. The two indicators of comprehensive park area and green area coverage.

**5. The results in Table 5 should be explained with more details, for example the difference between industry consumption and household consumption. Different city types may have different constitution of industry and household consumption and whether it causes the super-linear scaling in industrial cities, but not commercial and mixed cities.**

**Response:**

Based on your suggestions, we calculated the urban electricity consumption proportions in different types of cities and analyzed its possible impact on the scale of urban electricity consumption. The modifications are shown in the article.

**6. If you can access the electricity consumption data by different economic sector in each city, that would be an interesting perspective to see the scaling.**

**Response:**

We have worked hard to search the electricity consumption data of different economic sectors in various cities. This is indeed a very difficult thing because many data are missing. I'm sorry that this part of the content may not be supplemented in this research, but your suggestion is indeed very enlightening. It is a topic that is worth writing an article for in-depth research. We will try to fill this gap in the future.

**Specific comments:
line 180: how the 263 cities were selected, what are the relationship between 114 third-tier-and-above cities, 338 prefecture-level cities and 263 cities?**

**Response:**

China’s business charm rankings have divided 338 prefecture-level cities in China. We hope to conduct research within these cities. However, the China City Statistical Yearbook and China City Construction Statistical Yearbook do not include all 338 prefecture-level cities. At the same time, there are many data missing in the two yearbooks. After the cross-comparison and supplement of the data, only the data of 263 cities is relatively complete, so the data table containing 263 cities is used as the original data. 263 cities include all prefecture-level cities of different levels, and we only want to select more developed cities of third-tier and above to conduct research, so only 114 cities of third-tier and above are taken as the final research scope.

**line 205: why compare with 263 cities? Rather than 338 cities or 114 cities.**

**Response:**

The city type division method used in the study was conducted by comparing the proportions of different industries in each city with the national average. The 263 urban development data obtained are defaulted to represent the national average. So our compare is within the scope of the 263 cities, but not 338 or 114 cities.

**line 213: does the yearbook you mentioned provides all data at municipal districts level?**

**Response:**

Yes, these two yearbooks can provide all the data at the municipal level, among which some data are missing. We have filled in the data by consulting the provincial and municipal yearbooks and online databases.

**line 291: the sentence should be re-written to clarify which variables are individual needs, for example the drainage pipeline is individual needs or urban infrastructure?**

**Response:**

The table including the indicator name, unit, specific classification and data source has been included in the supporting information.

**line 336: “while urban water and water”**

**Response:**

I'm sorry, it was my fault. It should be “while urban water and electricity”. It has been modified. Sorry again.

**Reviewer #2:**

**1. It is suggested that the introduction should be reorganized. On the one hand, the content of urban crime mentioned in the introduction is not highly related to the article. On the other hand, the introduction discusses the size and scale of the urban, but at the end of the article, it is mentioned that the analysis is based on the type of urban, and the relationship between the two is not high.**

**Response:**

Based on your suggestions, we have made the necessary cuts and modifications to the introduction.

1. The part of the city crime not discussed in this article has been deleted.

2. The impact of city size on city development is an important part of our research. Using the urban population to represent the size of the city is an important prerequisite for the Bettencourt scale model. On this basis, we have increased the urban GRP to represent the economic scale of the city. Therefore, the scaling indexes of various urban development indicators with the urban population and urban GRP reflects the impact of urban size on urban development. City type is an important dimension of our research. Through separate analysis of different types of cities, it can help us understand the differences in scalig characteristics between different types of cities. Therefore, city size and city type are two dimensions of research, rather than two variables with low correlation.

**2. In this paper, the urban scaling characteristics are mentioned and the third-tier-and-above urban are selected for analysis. However, the analysis process of the full text is mostly based on the urban type, and there is less research on the urban scale. It is suggested to add comparative analysis of the characteristics of different scale of urban in the analysis, and the research value may be better.**

**Response:**

The answer to this question is similar to the answer to the previous question. The impact of city size on urban development is an important part of our research. Using the urban population to represent the size of the city is an important prerequisite for the Bettencourt scale model. On this basis, we have added urban GRP to represent the urban economic scale. Therefore, the scaling indexes of various urban development indicators along with urban population and urban GRP reflects the impact of urban size on urban development. City type is an important dimension of our research. Through separate analysis of different types of cities, it can help us understand the differences of scaling characteristics between different types of cities.

**3. The percentages of various indicators in different types of cities are compared and analyzed in Fig. 3, but it can be seen that there are great differences in the number of commercial cities, industrial cities and mixed-economy cities. Comparing the percentages of various cities with the percentages of consumed resources directly, the reliability and reliability of the results are not high. For comparative analysis, it is recommended to compare the average values of various cities.**

**Response:**

Based on your suggestions, we have modified this part of the content and compared the average of different types of cities with the overall average, which is indeed more intuitive.

**4. The article uses a lot of space to analyze the influencing factors of urban electricity consumption, which is not closely related to the theme of this article. Electricity consumption is an important indicator of urban scaling. The indicators ,such as the scale of urban construction land, urban population, water consumption, and transportation, can be used as key indicators that affect the size of the city. What are the reasons and particularities of analyzing the power consumption of the city?**

**Response:**

Urban electricity is not only a necessary energy for the development of urban commerce and industry, but also an important energy for the living of urban residents. More importantly, urban electricity consumption is also an important source of urban CO2 emissions. Therefore, electric energy consumption is closely related to the ecological environment. At the same time, compared with other indicators, the urban statistical yearbook provides a more detailed dimension of urban energy consumption data, which provides the possibility for in-depth analysis of urban energy consumption. In-depth analysis of urban electricity consumption can not only provide a reference for energy conservation, but also help relieve the pressure on the ecological environment.

**5. The conclusions mentioned in this paper can be used to formulate effective targeted policies for cities and to provide suggestions for reducing resource consumption in the process of urbanization. However, the end of this paper describes this part too little, and it is suggested to elaborate on different types or cities of different scales.**

**Response:**

According to your suggestion, we have supplemented the conclusion. For different types of cities, we have put forward different policy recommendations.

**Reviewer #3:**

**1.This manuscript analyse the urban scaling characteristics of China’s cites, the topic is interesting. However the novelty of this manuscript is lack. I did not see new findings on the urban scaling. The resluts of in figure 1 and 2 are not fresh knowledges.**

**Response:**

The law of urban expansion scale is the universal law of urban expansion established by Bettencourt based on the urban population, and it shows regularity without being restricted by space and time. China is rarely studied because of its complicated urbanization. We select more developed Chinese cities to try to verify whether the scaling law established by Bettencourt is applicable in China, and introduce urban GRP as an important independent variable. Although such research is not new, this universality provides a basis for China to master the urbanization process and make reference to the urbanization of other countries.

**2.For the indicators selection. the street lamp number was eclected as urban infrastructure indicator. In my opinions, the building and food supply are more important infrastructure than street lamp.**

**Response:**

The number of city street lights is closely related to the urban population and living environment, and is the most dense urban infrastructure. Urban street lights are often concentrated in areas with a high population density. The increase in the total number of street lights can reflect the population aggregation and infrastructure construction caused by urbanization. Therefore, the total number of urban street lights has been adopted by us as one of the infrastructure indicators. The city's architecture and food supply are relatively complex, which may involve many types of indicators. It may not be appropriate to conduct research directly as infrastructure, and it needs to be analyzed separately in subsequent studies.

**3.Figure 2 shows the results of relationship of natural gas supply pipe line with population, but in some provinces in China, there have no such natural gas pipe due to lack of this kind of resource。**

**Response:**

The data used in our research are all municipal districts. The natural gas pipeline facilities in more developed cities are relatively complete. China has also adopted the "west-to-east natural gas transmission" to complete the construction of pipeline facilities nationwide. According to our research results, it can be found that the urban natural gas pipeline shows a super-linear scaling relationship with the urban population, indicating that the construction of natural gas pipeline varies greatly among different cities, and that there is still much room for improvement in the construction of natural gas pipeline in Chinese cities.

**Reviewer #4:**
**1. Introduction is long enough, and it is suggested that a part of literature review can be added. such as "1. Introduction; 2.Literature review;…"**

**Response:**

We have cut and modified the introduction part of the article, but did not split it into two parts. In the introductory part, we hope to be able to tell the reader the background, research content, and significance of the article as if telling a story. Splitting the introductory part may cause the part to lose its integrity and be unclear. If you think there is still a problem with the revised introduction, we will modify it again according to your suggestions

**2.Research data needs descriptive statistical analysis of the data and more detailed explanation of the data source and processing.**

**Response:**

A table including the indicator name, unit, specific classification and data source has been included in the supporting information.

**3.According to "2016 China Business Charm Ranking", there are 119 third-tier-and-above cities, Why this article selected 114 third-tier-and-above cities ?**

**Response:**

According to the “2016 China Business Charm Ranking”, there are 119 third-tier cities and above in China, but the data of five of these cities (including three second-tier cities and two third-tier cities) are seriously missing or not included in China city statistical yearbook, so our study only adopt 114 third-tier cities and above.

**4.What are the 25 variables related to urban scaling and why are they chosen?**

**Response:**

The 25 variables associated with urban growth include:

population density, per capita road area, urban area, urban population, built-up area, urban construction land area, public facility land, fixed asset investment, street lights number, road area, park area, land area, GRP, per capita GRP, total water supply, electricity consumption for the whole society, industrial electricity, residential electricity, total gas supply, number of buses, operating vehicles, number of taxis, green area coverage and Three industries account for the proportion of GRP.

The 25 variables are all from China's urban statistical yearbook and China's urban construction statistical yearbook, covering most of the indicators of urban innovation wealth, material energy or infrastructure, which are also important factors to measure the level of urbanization. Therefore, we selected these indicators for analysis and calculation.

**5.If the research results can be compared with the existing research, the research contribution of this article will be enhanced.**

**Response:**

Thank you for your suggestions and we have supplemented the conclusion. Comparing our research results with previous studies can help readers understand the significance of the research.

We have tried our best to improve the manuscript and made some changes in the

manuscript. These changes will not influence the content and framework of the

paper. And here we did not list the changes but marked in revised paper.

We appreciate for editors and reviewers’ warm work earnestly and hope that the

correction will meet with approval.

Once again, thank you very much for your comments and suggestion.

Best regards,

Xingchao Liu and Zhihong Zou
