# Supplementary material for: Analysis on urban scaling characteristics of China’s relatively developed cities
Source: PLoS One. 2020 Jul 29;15(7):e0236593. doi: 10.1371/journal.pone.0236593 (PMC7390399; doi:10.1371/journal.pone.0236593)
Supplement: Attachment — Submitted filename: Response to Reviewers.doc [file pone.0236593.s003.doc]

Dear Editors and Reviewers:

Thank you for your letter and for the reviewers’ comments concerning our manuscript entitled “Analysis on urban scaling characteristics of China’s relatively developed cities” (ID: PONE-D-20-08745). Those comments are all valuable and very helpful for revising and improving our paper, as well as the important guiding significance to our researches. We have studied comments carefully and have made correction which we hope meet with approval.

The responds to the reviewer’s comments are as following.

**Responds to the reviewer’s comments:**

**Reviewer #1:**

I don't think one of my concerns, which is the small sample size for the 14 industrial cities has been addressed properly. My concern is from the perspective of statistics, saying that you need a decent sample size to conduct any regression analysis and to deliver valid results. Apparently 14 is not a decent size for regressions. The negative Adj-R^2^ in Table 2 also indicates that you have small R-square and a small sample size. One possible solution maybe the inclusion of panel data for the cities to increase the sample size.

**Response to Reviewer #1:**

According to your suggestion that we should increase the sample number of industrial cities, we selected 42 industrial cities for the calculation of the scaling index of urban development indicators with the change of urban population and urban GRP. The 42 industrial cities include not only 14 third-tier and above industrial cities, but also 28 industrial cities from the fourth and fifth tier.

Table 1 shows the scaling index results of the development indicators of 42 industrial cities with the change of urban population and urban GRP, and Table 2 shows the scaling index results of 14 third-tier and above industrial cities with the change of urban population and urban GRP.

From Table 1 and Table 2, it can be found that, even if the selection scope of industrial cities is expanded and the sample number of industrial cities is increased, the Adj-R^2^ obtained by 42 industrial cities is not significantly improved compared with the previous 14 industrial cities. In the regression results with urban population, Adj-R^2^ is mostly within the range of 0.1-0.3, which is still not a reliable R^2^ value. In the regression results with urban GRP, except that the R^2^ value of fixed asset investment has been significantly improved, the other R^2^ values are still at a low range level.

In fact, we can't find any more industrial cities in the third-tier and above.If we want to increase the sample size, we may need to include some underdeveloped industrial cities. However, this conflicts with our research topic, and does not result in more reliable regression results (R^2^ values did not increase significantly in 42 industrial cities).

The number of industrial cities in China is relatively small, and the development level and urbanization level of industrial cities are relatively low. The unremarkable scaling characteristics of industrial cities in the research results is more due to the lag of the development models of industrial cities. The development of industrial cities is more dependent on industry. Compared with commercial cities and mixed economy cities with a larger commercial proportion, they are less attractive to residents. The ecological environment deterioration caused by industrial development further intensifies the outflow of local residents. Therefore, urban physical energy indicators, infrastructure indicators and other development indicators that are closely related to residents have failed to perform well in proportion to the urban population. Therefore, the number of third-tier and above industrial cities in the study is not an important factor affecting the scaling results, but the development characteristics of industrial cities themselves are the main reason.

The development characteristics of China's industrial cities are quite different from those of other types of cities, suggesting that the Chinese government should take more measures in accordance with local conditions when making urban development policies.

In our study, industrial cities show very different development characteristics, which are often neglected in previous studies.It is worth further in-depth study to explore the development characteristics of industrial cities. We also hope to explore and analyze the scaling laws of industrial cities separately in the future, which is really an interesting and worthwhile subject.

**Table 1. Scaling index results for the development indicators of 42 industrial cities in China**

| City  type | LN(RV) | With respect to LN(Population) | | | With respect to LN(GRP) | | |
| --- | --- | --- | --- | --- | --- | --- | --- |
|  |  |  |  |  |  |  |  |
|  |  | b | Linearity | Adj-R^2^ | b(Std.Error) | Linearity | Adj -R^2^ |
| City-I  (n=42) | TGS | 0.787 | Sub-L | 0.064 | 0.811 | Sub-L | 0.135 |
|  | TWS | 0.509 | Sub-L | 0.103 | 0.850 | L | 0.58 |
|  | WSEC | 0.398 | Sub-L | 0.055 | 0.754 | Sub-L | 0.46 |
|  | FAI | 0.707 | Sub-L | 0.266 | 0.785 | Sub-L | 0.588 |
|  | CLA | 0.643 | Sub-L | 0.351 | 0.637 | Sub-L | 0.600 |
|  | DPL | 0.612 | Sub-L | 0.086 | 0.829 | Sub-L | 0.32 |
|  | RA | 0.502 | Sub-L | 0.160 | 0.665 | Sub-L | 0.523 |
|  | PA | 0.589 | Sub-L | 0.113 | 0.650 | Sub-L | 0.258 |
|  | SLN | 0.434 | Sub-L | 0.123 | 0.441 | Sub-L | 0.233 |

Table 2. Scaling index results for the development indicators of 14 third-tier and above industrial cities in China

| City  type | LN(RV) | With respect to LN(Population) | | | With respect to LN(GRP) | | |
| --- | --- | --- | --- | --- | --- | --- | --- |
|  |  |  |  |  |  |  |  |
|  |  | b | Linearity | Adj-R^2^ | b(Std.Error) | Linearity | Adj -R^2^ |
| City-I  (n=14) | TGS | -1.236 | - | 0.063 | -0.368 | - | -0.046 |
|  | TWS | -0.769 | - | -0.002 | 1.218 | Super-L | 0.502 |
|  | WSEC | 0.924 | L | 0.029 | 1.487 | Super-L | 0.674 |
|  | FAI | -0.287 | - | -0.072 | 0.062 | - | -0.082 |
|  | CLA | 0.078 | - | -0.08 | 0.750 | Sub-L | 0.601 |
|  | DPL | 0.432 | - | -0.06 | 0.982 | L | 0.269 |
|  | RA | -0.374 | - | -0.031 | 0.751 | Sub-L | 0.523 |
|  | PA | -0.127 | - | -0.079 | 0.380 | Sub-L | 0.025 |
|  | SLN | 0.536 | Sub-L | 0.031 | 0.326 | Sub-L | 0.038 |

We appreciate for editors and reviewers’ warm work earnestly and hope that the response will answer the reviewer’s question.

Once again, thank you very much for your comments and suggestion.

Best regards,

Xingchao Liu and Zhihong Zou
